# Prevalence of HCV NS3 pre-treatment resistance associated amino acid variants within a Scottish cohort

**DOI:** 10.1016/j.jcv.2015.02.005

**Published:** 2015-04

**Authors:** Samantha J. Shepherd, Tamer Abdelrahman, Alasdair R. MacLean, Emma C. Thomson, Celia Aitken, Rory N. Gunson

**Affiliations:** a West of Scotland Specialist Virology Centre, Level 5, New Lister Building, 10-16 Alexandra Parade, Glasgow G31 2ER, United Kingdom; b MRC–University of Glasgow Centre for Virus Research, Stoker Building, 464 Bearsden Road, Glasgow G61 1QH, United Kingdom

**Keywords:** Simeprevir, HCV, Protease inhibitor, Prevalence, RAV

## Abstract

**Background:**

Protease inhibitors (PI) including boceprevir, telaprevir and simeprevir have revolutionised HCV genotype 1 treatment since their introduction. A number of pre-treatment resistance associated amino acid variants (RAVs) and polymorphisms have been associated with reduced response to treatment.

**Objectives:**

We measured the prevalence of RAVs/polymorphisms in a PI treatment-naïve HCV genotype 1 Scottish cohort using Sanger sequencing.

**Study design:**

Chronically infected, treatment-naïve, HCV genotype 1 patients (*n* = 146) attending NHS Greater Glasgow and Clyde clinics were investigated for RAVs/polymorphisms to the PIs boceprevir, telaprevir and simeprevir. The NS3/4A region was amplified by nested polymerase chain reaction. The 1.4 kb amplified product was sequenced using an ABI 3710XL DNA sequencer. Sequence analysis was performed using web-based ReCall (beta 2.10). Amino acid positions 36, 41, 43, 54, 55, 80, 109, 122, 155, 156, 168 and 170 were analysed for RAVs/polymorphisms.

**Results:**

Overall, 23.29% (34/146) of patients had an RAV or polymorphism detected. Overall, 13.69% (20/146) of patients had HCV virus that contained the Q8 K polymorphism. Other RAVs detected were: V36 M 0.70% (1/146), V36L 0.70% (1/146), T54S 6.85% (10/146), V55A 3.42% (5/146) and V/I170A 0.68% (1/146). Four patients had dual combinations of mutations (T54S + V36L; T54S + V55A and 2 patients with T54S + Q80K).

**Conclusions:**

Q80K was the most prevalent baseline polymorphism detected in the Scottish cohort. Simeprevir treatment is not recommended in patients infected with the Q80K genotype 1a variant. This highlights the need for baseline sequencing prior to administration of this drug in this population.

## Highlights

• Prevalence of HCV NS3 resistance-associated amino acid variants (RAV) was investigated.
• Sanger sequencing was performed on a protease inhibitor treatment-naïve Scottish cohort (*n* = 146).
• Overall 23.29% had a detectable RAV in the NS3 region; Q80K was the most prevalent.
• Only 2.74% patients had more than one RAV.
• The study highlighted the need for Q80K sequencing prior to simeprevir treatment.

## 1 Background

Traditionally genotype 1HCV infections have been the hardest to treat with sustained virological response (SVR) rates to the standard of care treatment of ribavirin (RBV) co-administered with pegylated-interferon alpha (IFN) in the region of 42–50% [1], [2]. The development of non-structural protein 3 (NS3) protease inhibitors (PIs) including telaprevir, boceprevir and simeprevir, has substantially improved outcome in these patients with SVR rates now approaching 80% in both treatment-naive patients and relapsers [3], [4], [5], [6]. Newer PI based IFN-free regimens show even greater potential and lower toxicity. For example, the combination of simeprevir and the NS5B polymerase inhibitor sofosbuvir increased the SVR to over 90% in genotype 1 patients [7]. NS5A inhibitors daclatasvir or ledipasvir when used with sofosbuvir have also generated SVR rates >90% [8], [9]. Further breakthroughs are expected as other combinations of antivirals become available which promise to offer improvements in SVR, shortened duration of treatment and lower pill burden.

A number of pre-treatment resistance associated amino acid variants (RAVs) within NS3 are associated with reduced response to PI–IFN regimens. For example, RAVs at position 156 (A156T/V) and R155K have been shown to reduce the effectiveness of all current PIs [10], [11], [12], [13]. Substitutions at the D168 locus (D168T/Y/H/A/V/I) result in high-level resistance to simeprevir (>300 fold) and the other 2nd generation PIs only [13], [14], [15]. Resistance polymorphisms Q80K or R have been shown to negate the benefit of adding simeprevir to pegylated IFN and RBV [16] and, for this reason, it is recommended in the license that patients infected with genotype 1a HCV who have evidence of Q80K/R mutations are not considered for treatment with simeprevir. RAVs at amino acid positions 36, 41, 43, 54, 55, 109, 122 and 170 have also been reported [11], [13], [14], [17], [18]. However, their significance is currently uncertain with most reports suggesting that they only have a minor effect on overall SVR rates. Only a few studies have examined the prevalence of the aforementioned RAVs at baseline [19], [20], [21], [22], [23]. Knowing their frequency, can be used to plan treatment policies and will determine the usefulness of baseline testing prior to treatment.

## 2 Objectives

We measured the prevalence of natural resistance polymorphisms in a protease inhibitor treatment-naïve HCV genotype 1 Scottish cohort using Sanger sequencing.

## 3 Study design

### 3.1 Patients

Stored plasma samples, taken between August 2013 and March 2014 for 146 chronically infected HCV genotype 1 patients attending clinics within NHS Greater Glasgow & Clyde, were used in this study. The patients consisted of 141 treatment naïve patients and 5 treatment relapsers who had previously been treated with pegylated IFN and RBV. The majority of the patients (*n* = 140) were subtype 1a and six subtype 1b. All patients had a detectable HCV RNA tested by Abbott RealTime HCV (detection limit 12 IU/ml).

### 3.2 RNA extraction

RNA was extracted using the NucliSens easyMag (BioMerieux). Using the on-board lysis protocol, 1000 μl of sample was eluted to 60 μl.

### 3.3 PCR amplification and sequencing procedure

The NS3/4A region was amplified by nested polymerase chain reaction (PCR) using a method and primers supplied by Dr Richard Harrigan (British Columbia Centre for Excellence in HIV/AIDS). The 1st round primer sequences were: 5′ TTCAGCCTGGACCCTACCTTTACCAT 3′ (position 4731–4756), 5′ ATGGAGATCAAGGTCATCACGTGGGG 3′ (position 3276–3301) and 5′ GTGGCCGTAGAGCCTGTCGTCTTC 3′ (position 3246–3269). The 2nd round primer sequences were: 5′ GACTTCGACTCTGTGATAGACTGCAAC 3′ (position 4680–4706), 5′ TCAAGGTCATCACGTGGGGGGCGGA 3′ (position 3283–3307) and 5′ TACCGGCGACTTCGACTCGGTGAT 3′ (position 4673–4696). The 1st round PCR amplification was carried out using a Qiagen OneStep RT-PCR kit and the 2nd round with the Expand High Fidelity PCR system (Roche Diagnostics GmbH). Sanger sequencing was performed on the ABI 3710XL DNA sequencer with Big Dye v3.1. The 1.4 kb sequence was analysed using web-based ReCall beta v2.10 (http://pssm.cfenet.ubc.ca/home/index). Using the amino acid feature on ReCall, the following amino acid positions were analysed: 36, 41, 43, 54, 55, 80, 109, 122, 155, 156, 168 and 170.

## 4 Results

There was no evidence of RAVs at position 155, 156 and 168 in any of the sequences analysed (Table 1). The polymorphism Q80K was found in 13.69% (20/146) of patients sequenced. Other RAVs were found at the following frequencies: 0.70% (1/146) V36M, 0.70% (1/146) V36L, 6.85% (10/146) T54S 3.42% (5/146) V55A and 0.68% (1/146) V/I170A. Four patients were identified as having dual combinations of mutations (T54S + V36L; T54S + V55A and 2 patients with T54S + Q80K).

## 5 Discussion

This study analysed sequences from the NS3/4A serine protease region of 146 genotype 1 patients (140 were genotype 1a and 6 were genotype 1b). The low level of subtype 1b patients in this study is a reflection of the Scottish population, where subtype 1a predominates. This is also a reflection of the UK population as a whole [24]. Overall 23.29% of patients tested had NS3 RAVs/polymorphisms without prior exposure to PIs. No high-level resistant RAVs were detected at positions 155, 156 or 168. Other prevalence studies in treatment-naive patients have shown that these three key resistance mutations either occur at a very low level (<0.9%) or not at all [21.25,26]. The majority of patients had the naturally occurring polymorphism Q80K (13.69%). The prevalence of Q80K in the Scottish cohort is similar to that found in other European studies; France 10.5%; Italy 10.1%; London 16% and Sweden 5.7% [20], [22], [23], [27]. Q80K prevalence in the USA has been reported at higher prevalence levels of 37% and 47% [19], [21]. Mutational differences between genotype 1 subtypes and clades within subtype 1 may reflect differences seen between American and European patients [19], [28], [29]. Studies have also highlighted that Q80K is more likely to occur in patients with subtype 1a HCV than subtype 1b [20], [21].

The V36L/M, T54S, V55A and V/I170A mutations detected in this study are low level resistance RAVs that have little effect on SVR rates in patients treated with triple therapy [21], [30]. These indeterminate or low level RAVs have been reported at a prevalence of between 0.2% and 11% [20], [21], [25], [26], [27]. The mutations V36M, V36L and V/I170A do not appear to be detrimental to viral fitness compared with high level resistance mutations and may explain the presence of these mutations within untreated populations [10], [31]. In this study, T54S was found at a prevalence of 7.53% within the Scottish cohort. This mutation confers low level resistance to both boceprevir and telprevir but not simeprevir [13], [32], [33]. T54S has been identified in 7.5% treatment-naive patients in Sweden and 2.8% in Italy [20], [22].

In this study four (2.74%) subtype 1a patients were found to have RAV combinations, which all contained T54S with another mutation (T54S + V36L; T54S + V55A and T54S + Q80K). Bae et al. [19] found that the combination of T54S and Q80K did not increase drug resistance to simeprevir but did reduce resistance to boceprevir and teleprevir when compared to the single mutation T54S (<3 fold–5 fold). The combination of V36L + T54S has been reported previously [20]. It is unclear if this combination substantially increases resistance to PIs but the mutational combination of V36M + T54S increases viral fitness compared to a virus with T54S only [18]. Combination RAV at positions 54 and 55 have been shown to reduce response to triple therapy containing boceprevir [34].

This study examined the frequency of NS3 variants detected by Sanger sequencing. Sanger sequencing will only detect these variants at a frequency of >20%. Next generation sequencing (NGS) can detect lower frequency variants by measuring population variants that occur at <1% [35]. As a result studies using NGS will likely detect RAVs at an increased frequency. NGS studies of the NS3 region in treatment-naive patients, have again identified Q80K as the most prevalent baseline mutation with ∼42% harbouring this polymorphism [36], [37].

Currently, European guidelines have six suggested treatment options for genotype 1 patients with simeprevir recommended within two of these treatment protocols [38]. This study confirms that high-level resistance RAVs 155, 156 and 168 are rare within the treatment-naïve population in the West of Scotland. However, Q80K is common (13.69%) and baseline sequencing prior to therapy should be considered when considering simeprevir/IFN treatment in genotype 1a patients. It is possible that such testing will only be a temporary measure since newer dual therapies may largely overcome the negative effect of the Q80K mutation [39], [40].

## Funding

Janssen Pharmaceutical funded the implementation of Q80K testing in the West of Scotland Specialist Virology Centre. TA is funded by the MRC (G0801822) and ECT by the Welcome Trust (WT102789).

## Competing interests

None declared.

## Ethical approval

Not required.

## Figures and Tables

**Table 1:** NS3/4A mutations detected in a group of PI treatment naive patients. Mutation list was adapted from Lenz et al. [13]; Leggewie et al. [27]; Forns et al. [6]; Povada et al. [29]; Schneider and Sarrazin [41].

Amino acid position	Mutation	Prevalence in Scottish cohort (*n* = 146)	Drug
V36	M L G	1/146 (0.68%) 1/146 (0.68%) 0.00%	Boceprevir, telaprevir, simeprevir
Q41	R	0.00%	
F43	S I V	0.00% 0.00% 0.00%	Simeprevir
T54	S A	10/146 (6.85%) 0.00%	Boceprevir, telaprevir, simeprevir
V55	A	5/146 (3.42%)^a^	Boceprevir, telaprevir
Q80	K R	20/146 (13.69%) 0.00%	Simeprevir
R109	K	0.00%	Boceprevir
S122	R	0.00%	Simeprevir
R155	K T	0.00% 0.00%	Boceprevir, telaprevir, simeprevir
A156	S T V	0.00% 0.00% 0.00%	Boceprevir, telaprevir, simeprevir
D168	A V I T H E	0.00% 0.00% 0.00% 0.00% 0.00% 0.00%	Simeprevir
V/I170	A	1/146 (0.68%)	Boceprevir, telaprevir

a 1/5 of the sequences was a wild type/resistant mixture (V55A/V).
